# Programmed Adult Kidney Disease: Importance of Fetal Environment

**DOI:** 10.3389/fphys.2020.586290

**Published:** 2020-09-25

**Authors:** Rogério Argeri, Fernanda Thomazini, Débora Conte Kimura Lichtenecker, Karina Thieme, Maria do Carmo Franco, Guiomar Nascimento Gomes

**Affiliations:** ^1^Department of Physiology, School of Medicine, Federal University of São Paulo, São Paulo, Brazil; ^2^Department of Physiology and Biophysics, Institute of Biomedical Sciences, Universidade de Sao Paulo, São Paulo, Brazil

**Keywords:** renal function, programmed kidney disease, hypertension, nephron number, fetal environment

## Abstract

The Barker hypothesis strongly supported the influence of fetal environment on the development of chronic diseases in later life. Multiple experimental and human studies have identified that the deleterious effect of fetal programming commonly leads to alterations in renal development. The interplay between environmental insults and fetal genome can induce epigenetic changes and lead to alterations in the expression of renal phenotype. In this review, we have explored the renal development and its functions, while focusing on the epigenetic findings and functional aspects of the renin-angiotensin system and its components.

## Introduction

The number of nephrons in humans is highly variable, ranging from 250,000 to 2 million per kidney (Bertram et al., 2011). There is an inverse correlation between the total number of nephrons and the risk of developing kidney disease and hypertension (Brenner et al., 1988; Keller et al., 2003). Nephrons are composed of specialized cells, such as epithelial, endothelial, and stromal cells. The epithelial cells originated from the intermediate mesoderm, which also originates two different progenitor cell populations: the ureteric bud (UB) and the metanephric mesenchyme (Little et al., 2007). Clusters of metanephric mesenchymal cells condense around each ureteric bud to form cap mesenchyme (CM). Then, follows a complex and highly coordinated process that depends on interactions between the UB (which will form the collecting duct system) and surrounding CM (which originates the intermediate progenitor cells that ultimately generate all cell types of the nephron; Costantini and Kopan, 2010; Little and McMahon, 2012; Nicolaou et al., 2015; Hurtado Del Pozo et al., 2018). Grobstein’s study was a pioneer in renal development. It showed that kidney development is a multi-stage process, which starts with a primary induction event from the UB, followed by mesenchymal-to-epithelial transition within the CM. The process ends with the completion of nephron patterning and elongation (Grobstein, 1956).

## Kidney Disease and Barker Hypothesis: Importance of Fetal Environment

Renal disease is one of the common causes of mortality and morbidity worldwide (Luyckx et al., 2018). Interestingly, this disease can originate in early life (Hoy et al., 1999; Lackland et al., 2000; Eriksson et al., 2018). Barker proposed that insults during critical periods of fetal development can result in a growth deficit, characterized by low birth weight (LBW) and silent morpho-functional changes, that translate into kidney disease in the long term (Hales and Barker, 2001; Langley-Evans et al., 2003).

The human nephrogenesis requires an optimum balance and is completed by around 32–34 weeks of gestation. During this period, the kidneys can be influenced by insults in fetal environment. Evidence suggests that people with LBW have congenital deficit in the number of nephrons and are more susceptible to subsequent renal injury and functional decline in later life (Brenner et al., 1988; Schreuder et al., 2006; Eriksson et al., 2018). Reports that evaluated fetal kidney by ultrasound have supported these findings (Konje et al., 1996; Spencer et al., 2001). Interestingly, other studies have described a positive correlation between birth weight and number of nephrons (Feig et al., 2004; Hoy et al., 2005). Besides, an inverse correlation between the birth weight and glomerular size in the kidney, isolated from neonates, was observed (Mañalich et al., 2000). According to Brenner’s theory (Brenner et al., 1988), the reduced number of nephrons limits excretion of fluids and electrolytes, leading to volume expansion and development of hypertension; this alteration, in turn, damages the glomeruli, causing glomerulosclerosis and accelerating the loss of nephrons (Brenner et al., 1988; Hinchliffe et al., 1991; Schreuder et al., 2006). Conversely, the reduction in nephron number, by the reduction in renal mass or of congenital origin, is followed by compensatory renal growth that leads to hypertrophy of both glomeruli and tubules. This structural change, in addition to increasing glomerular filtration, results in the proximal and distal tubules growth favoring the reabsorption of the filtered volume. Thus, renal function is preserved; however, in long term may result in hypertension and kidney damage (Fong et al., 2014; McArdle et al., 2020).

The assessment of renal function is important to detect the extent and progression of renal diseases. Increasing evidence is available about the impact of fetal environment on the decline in renal function (Hoy et al., 1999; Giapros et al., 2007). An inverse correlation has been observed between birth weight and albumin-to-creatinine ratio in individuals from an Australian aboriginal community (Hoy et al., 1998). Other reports have observed a relationship between LBW with lower glomerular filtration rate (GFR) and albuminuria (Hoy et al., 1999, 2005; Keijzer-Veen et al., 2005). It has been described that LBW neonates, as a result of either prematurity or growth restriction, have increased levels of albuminuria (Aisa et al., 2016). Additionally, severe tubular injury, characterized by high levels of cathepsin B and N-acetyl-β-D-glucosaminidase (NAG) activity, was observed in these neonates (Aisa et al., 2016). In a twin study, Gielen et al. (2005) found that LBW population had lower creatinine clearance levels than the high birth weight population. Moderate renal disturbances were found in LBW children from 4 to 12 years of age (Monge et al., 1998). Another study has reported high levels of cystatin C, a marker of renal function, in LBW children (Franco et al., 2008b). Additionally, Barbati et al. (2016) have shown high levels of cystatin C in urine of LBW neonates. These authors observed an inverse correlation between cystatin C levels and renal volume (Barbati et al., 2016).

Several experimental studies were conducted to confirm the human data (Swanson and David, 2015). Studies demonstrated that restricted protein intake during pregnancy led to LBW, reduced nephron number, and glomerular enlargement in offspring (Merlet-Bénichou et al., 1994; Langley-Evans et al., 1999; Woods, 2000; Vehaskari et al., 2004; Mesquita et al., 2010). Additionally, protein restriction also increased: blood urea, urinary output, and urinary albumin excretion in resultant offspring (Nwagwu et al., 2000). It is believed that the renal changes occur in response to inadequate nutrition as adaptations to ensure survival (Langley-Evans, 2015). These adaptations would be adequate if in the postnatal period the nutritional conditions remained the same. However, if in the postnatal period the nutritional offer changes to a better nutritional standard, the occurred adaptations are no longer adequate and become harmful. This discrepancy between the phenotype developed and that suitable for a given environment is called “mismatch” (Gluckman et al., 2019).

Another important prenatal factor contributing to programming of the renal diseases is maternal diabetes mellitus (e.g., pre-existing type 1 or 2 and gestational diabetes). Although it is correlated with high, instead of LBW, is also associated with impairment of both GFR and renal plasma flow (Abi Khalil et al., 2010). The effect of maternal diabetes on fetal kidney volume is not well established (Hokke et al., 2019). In a recent study, instead of nephron number, the total renal and cortical volumes were assessed in newborns. Neonates of diabetic mothers, with inadequate glycemic control, had lower cortical and total renal volume, suggesting a lower nephron number. However, newborns of diabetic mothers, with strict glycemic control, had similar values as control newborns (Aisa et al., 2019). Thus, it is evident that variations in the maternal glycemic status can lead to different outcomes in the offspring’s kidneys (Aisa et al., 2019).

The studies about the impact of maternal diabetes on fetal development are of great importance since this metabolic disorder can promote several congenital anomalies (Steel et al., 1990). Some studies have demonstrated a high risk of congenital anomalies of the kidney and urinary tract (CAKUT) in children exposed to maternal diabetes (Shnorhavorian et al., 2011; Hsu et al., 2014). Dart et al. (2015) reported that pre-gestational diabetes increased the risk of CAKUT in infants by 67%. Maternal hyperglycemia is an important threat, as glucose crosses the placenta leading to fetal hyperinsulinemia (Higgins and Mc Auliffe, 2010). In turn, hyperinsulinemia stimulates leptin secretion, resulting in hyperleptinemia (Lepercq et al., 1998). Fetal exposure to non-physiological concentrations of these hormones is associated with hypothalamic dysfunction (Plagemann, 2004). Additionally, generation of reactive oxygen species can occur in response to increased glucose/insulin metabolism (Tozour et al., 2018). Therefore, maternal hyperglycemia can be threatening to the developing fetus by different mechanisms. Hence, it is crucial to maintain optimum blood glucose levels during pregnancy.

Experimental studies in female rats, in which diabetes mellitus was induced before the onset of pregnancy using streptozotocin (STZ), demonstrated that offspring developed glomerular hypertrophy and reduction of both GFR and urinary output (Rocha et al., 2005; Magaton et al., 2007). Additionally, renal vascular resistance and the thickness of interlobular arteries were increased in the offspring of diabetic mothers, suggesting vascular remodeling (Rocco et al., 2008); results recently confirmed by Dib et al. (2018). However, there are controversies regarding the nephron number in offspring of diabetic mothers (Rocha et al., 2005; Magaton et al., 2007; Rocco et al., 2008). Reduction in nephron number was observed in studies in which diabetes was induced by different protocols after confirmation of pregnancy (Amri et al., 1999; Tran et al., 2008; Hokke et al., 2013). However, in these studies, the fetus was exposed to the direct effects of STZ, in addition to hyperglycemia.

## Mechanisms Involved in Programmed Kidney Diseases

**Figure 1:**
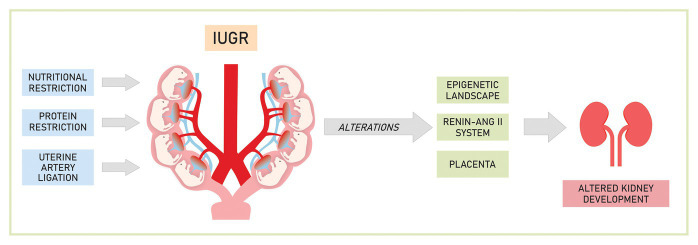
Mechanisms involved in the development of renal changes caused by experimental intrauterine growth restriction (IUGR).

Many are the possible mechanisms involved in fetal programming, in Figure 1 are represented the most studied ones.

### Epigenetic Events

The fetal environment can alter the epigenetic landscape, strongly impacting the reprogramming of gene expression (Dressler and Patel, 2015). Epigenetic modification refers to dynamic changes in chromatin that mediates interactions between environmental factors and the genome. These modifications alter chromatin structure, thereby modulating the accessibility of transcription factors and consequently determining the expression or repression of genes (Hurtado Del Pozo et al., 2018; Wanner et al., 2019). Some of the well-known epigenetic modifications are DNA methylation and post-translation modifications of histones (acetylation, methylation, phosphorylation, and ubiquitination; Hilliard and El-Dahr, 2016).

The role of DNA methylation in the nephron development and its function is not yet well defined. Recently, Wanner et al. (2019) reported that DNA methyltransferase 1 (Dnmt1), and not Dnmt3a/b, is the key regulator of prenatal renal programming, representing a fundamental link between nephron number and intrauterine environment. On the other hand, histone marks distinctively dictate nephrogenesis and affect the expression of several developmental genes, some of them involved in renal disease development (Harikumar and Meshorer, 2015). Studies have provided important information about spatiotemporal changes and dynamic functional states of the epigenome during nephrogenesis. These studies have demonstrated that promoter regions of specific genes in nephron progenitor cells are bivalent and carry both “active” (H3K4me3) and “repressive” (H3K9me3 and H3K27me3) histone marks, along with expression of their corresponding methyltransferases (Ash2l, G9a, Ezh2/Suz12, respectively; McLaughlin et al., 2013, 2014). This suggests that differentiation programs are silenced in these cells, however, can be activated in response to external factors that can be determined by maternal nutritional features during fetal life. Dnmt1 and H3K27me3 are critical in nephron progenitor cells (NPCs) self-renewal and differentiation (Huang et al., 2020). NPC express H3K4me3/K9me3/K27me3 but nascent nephrons retain H3K4me3 marks and downregulate H3K9me3/K27me3 (Hilliard and El-Dahr, 2016). Remarkably, alterations in H3K27me3 levels in differentiated cells have been linked to renal diseases (Majumder et al., 2018; Jia et al., 2019).

Gene-environment interactions during fetal development can persist in multiple generations. Some studies have reported that *in utero* exposure can affect fetal germ cells and F2 offspring, resulting in transgenerational programming. Epigenetic changes at birth can be associated with maternal nutrition and in the case of female fetuses they can be transmitted to the next generation, emphasizing that inheritance does not depend only on genetic factors but also involves epigenetic mechanisms (Gluckman et al., 2019). However, still little is known about these effects on kidney disease (Briffa et al., 2020).

### Renin-Angiotensin System

It is well established that the renin-angiotensin system (RAS) plays an important role in normal morphological development of the kidney and renal function (Guron and Friberg, 2000). All the components of RAS are expressed in early gestation in both rats and human beings (McMillen and Robinson, 2005). In fact, the pharmacological blockade of RAS during renal development leads to altered renal structures (Machado et al., 2008; Madsen et al., 2010; Kett and Denton, 2011; de Almeida et al., 2017). Mutations and altered expressions in genes coding for RAS components are linked to congenital abnormalities of the kidney, confirming the importance of RAS in renal morphogenesis and metanephric organogenesis (Song et al., 2011). It was demonstrated that angiotensin II stimulates the growth and branching of the UB through its receptors (Yosypiv et al., 2008; Song et al., 2011). Also, angiotensin II stimulates the expression of Pax-2 (homeobox 2 gene), an anti-apoptotic factor, which is essential for renal development and repair (Hershkovitz et al., 2007). This process occurs through AT_2_ receptors (AT2r) and modulates nephrogenesis and renal development (Zandi-Nejad et al., 2006).

The expression of some RAS components could be sensitive to insult during fetal development contributing to later development of renal diseases. Evidence in the literature has demonstrated that fetal programming by several insults promote suppression of the fetal RAS, resulting in altered nephrogenesis (Woods et al., 2001; Dötsch et al., 2009; Mesquita et al., 2010; Moritz et al., 2010). Woods et al. (2001) showed reduction in messenger RNA (mRNA) expression and renin levels in newborns exposed to low protein diet in prenatal life. These authors suggest that such early alteration may be caused by lower intrarenal angiotensin II concentration during nephrogenesis and consequently impair the renal development.

It was observed that fewer ureteral branches sprouted out from the metanephros in the fetus exposed to low protein diet in utero (Mesquita et al., 2010). These changes occurred simultaneously with decreased expression of AT1 receptors (AT1r; on the 17th embryonic day), confirming maladjustment of RAS in the kidneys of these animals (Mesquita et al., 2010).

On the other hand, after the end of nephrogenesis, hyperactivity of RAS was observed in response to fetal programming. There is evidence that exposure to low protein diet, throughout gestation or during specific periods, results in an imbalance in the expression of AT1r and AT2r (Moritz et al., 2010). An increase in AT1r mRNA and protein and a decrease in AT2r gene expression were observed (McMullen et al., 2004; Vehaskari et al., 2004; McMullen and Langley-Evans, 2005). Moreover, it was reported high expression of both mRNA and proteins coding for Angiotensin II 1b receptor (AT_1_b-R) in adrenal glands isolated from adult offspring exposed to protein restriction during fetal life; and also, hypo-methylation in the promoter regions ofAT_1_b-R gene (Bogdarina et al., 2007). This epigenetic alteration could lead to higher transcriptional activity, thereby promoting higher expression of this receptor.

Considering maternal diabetes models, it has also been described that the expression of the mRNA coding for angiotensin 1–7 was significantly lower in the offspring of diabetic mothers (Magaton et al., 2007). Besides, increased expression of AT1r was observed in the kidneys of offspring from diabetic mothers, suggesting that changes in RAS might have contributed to the renal changes in this model (Yan et al., 2014).

Interestingly, the RAS blockade in early post-natal life seems to offset the negative effects of fetal programming on the kidneys (Manning and Vehaskari, 2005; Hsu et al., 2015; Watanabe et al., 2018). Early administration of aliskiren, a renin inhibitor, reduced angiotensinogen expression associated with increased renal AT2r and Mas protein in offspring exposed to nutrient restriction during gestation (Hsu et al., 2015). Also, post-weaning losartan (AT1r blocker) therapy completely stopped immune cell infiltration and intrarenal RAS activation in the kidneys isolated from adult offspring exposed to protein restriction during fetal life (Watanabe et al., 2018).

Additionally, altered expression of AT1r in areas of the brain involved in the regulation of blood pressure has been found in protein-restricted offspring (Pladys et al., 2004; de Lima et al., 2013). This change is probably associated with the autonomic changes found in these animals. Increased sympathetic tone seems to have an important role in the genesis of arterial hypertension in this experimental model, supporting this hypothesis are the high plasma concentration of catecholamines observed in protein-restricted offspring (Petry et al., 2000); and the fact that renal denervation prevented hypertension confirming the role of renal nerves activation in the offspring with restricted growth (Alexander et al., 2005; Custódio et al., 2017).

Regarding the changes in RAS in humans, Simonetti et al. (2008) observed higher salt sensitivity in LBW children, possibly as a result of increased aldosterone activity or alterations in AT1r expression or affinity. It has also been reported that circulating levels of angiotensin II and angiotensin-converting enzyme (ACE) activity were higher in healthy males with LBW (Franco et al., 2008a). Ajala et al. (2012) found that the presence of DD genotype in the ACE gene is associated with higher ACE activity in children with a history of LBW. Moreover, one study in LBW children has reported hypo-methylation in the promoter region of the ACE gene (Rangel et al., 2014). These authors also observed that LBW children have lower methylation levels along with higher ACE activity (Rangel et al., 2014).

### Placental Alterations

The placenta, which forms the functional interface between the maternal and fetal circulations, is important for the normal development of the fetus. Usually, the placenta expresses the enzyme 11 beta-hydroxysteroid dehydrogenase type 2 (11β-HSD2) that inactivates maternal glucocorticoids, protecting the fetus from premature exposure to this hormone. Studies have shown that maternal nutritional restriction significantly reduces placental development and also the expression of 11β-HSD2, thus the fetus may be exposed to these hormones prematurely (Börzsönyi et al., 2012; Chapman et al., 2013).

In rats, premature exposure to corticosterone impacted kidney formation resulting in reduction of the nephron number, confirming the negative effects of early exposure to this hormone (Woods and Weeks, 2005; Singh et al., 2007).

## Conclusion

Kidney disease is a concerning health problem in the modern world and can be considered as one of the common causes of morbidity and mortality. The fetal environment is an important period for the development of several adaptive mechanisms in various organ systems, leading to an increased risk of development of renal diseases in later life. Several reports from human and animal models suggest that the kidneys can be influenced by insults during fetal development in utero. The impairment in nephrogenesis leads to the process of glomerulosclerosis and loss of renal function in later life. Modifications in the epigenetic characteristics and RAS components are likely involved. Further studies are required to determine relationships between epigenetic alterations and RAS pathway abnormalities, and their ability to influence fetal programming of the kidney diseases.

## Author Contributions

All authors listed have made a substantial, direct and intellectual contribution to the work, and approved it for publication.

### Conflict of Interest

The authors declare that the research was conducted in the absence of any commercial or financial relationships that could be construed as a potential conflict of interest.
